# Systematic characterization of seminal plasma piRNAs as molecular biomarkers for male infertility

**DOI:** 10.1038/srep24229

**Published:** 2016-04-12

**Authors:** Yeting Hong, Cheng Wang, Zheng Fu, Hongwei Liang, Suyang Zhang, Meiling Lu, Wu Sun, Chao Ye, Chen-Yu Zhang, Ke Zen, Liang Shi, Chunni Zhang, Xi Chen

**Affiliations:** 1 State Key Laboratory of Pharmaceutical Biotechnology, Collaborative Innovation Center of Chemistry for Life Sciences, Jiangsu Engineering Research Center for MicroRNA Biology and Biotechnology, NJU Advanced Institute for Life Sciences (NAILS), School of Life Sciences, Nanjing University, 163 Xianlin Road, Nanjing, Jiangsu, 210046, China; 2 Department of Clinical Laboratory, Jinling Hospital, Clinical School of Medical College, Nanjing University, 305 East Zhongshan Road, Nanjing, Jiangsu, 210002, China; 3 Department of andrology, Affiliated Drum Tower Hospital of Nanjing University Medical School, 321 Zhongshan Road, Nanjing, Jiangsu, 210008, China; 4 Key Laboratory of Cancer Prevention and Therapy, Tianjin Medical University Cancer Institute and Hospital, Huanhuxi Road, Tiyuanbei, Tianjin, 300060, China

## Abstract

Although piwi-interacting RNAs (piRNAs) play pivotal roles in spermatogenesis, little is known about piRNAs in the seminal plasma of infertile males. In this study, we systematically investigated the profiles of seminal plasma piRNAs in infertile males to identify piRNAs that are altered during infertility and evaluate their diagnostic value. Seminal plasma samples were obtained from 211 infertile patients (asthenozoospermia and azoospermia) and 91 fertile controls. High-throughput sequencing technology was employed to screen piRNA profiles in seminal plasma samples pooled from healthy controls and infertile patients. The results identified 61 markedly altered piRNAs in infertile patient groups compared with control group. Next, a quantitative RT-PCR assay was conducted in the training and validation sets to measure and confirm the concentrations of altered piRNAs. The results identified a panel of 5 piRNAs that were significantly decreased in seminal plasma of infertile patients compared with healthy controls. ROC curve analysis and risk score analysis revealed that the diagnostic potential of these 5 piRNAs to distinguish asthenozoospermic and azoospermic individuals from healthy controls was high. In summary, this study identifies a panel of piRNAs that can accurately distinguish fertile from infertile males. This finding may provide pathophysiological clues about the development of infertility.

Infertility remains a prevalent health problem worldwide that affects 8–12% of reproductive-aged couples^1^, and male causes for infertility are found in approximately half of childless couples^2^,^3^. There are many pathogenic factors that lead to male infertility, but approximately 60~75% of infertility cases are idiopathic^4^. At present, the diagnosis of male infertility has mainly been based on traditional semen parameters, including seminal volume, pH, sperm concentration, motility and morphology, as recommended by the WHO^5^. Semen is a viscous mixture of spermatozoa and fluid from the seminiferous tubules, epididymis and accessory glands. Because semen can be obtained with relative ease, it is reasonable to search for noninvasive seminal biomarkers. However, several studies have demonstrated that routine semen analysis has failed to accurately distinguish between fertile and infertile males^4^,^6^. More importantly, traditional methods focus on macroscopic and superficial examinations and may ignore the internal causes of male infertility. Thus, it is important to search in semen for new biomarkers that have a high specificity and sensitivity for male infertility and also to provide additional information about the molecular mechanism underlying this condition.

Recent studies by us and other groups have demonstrated that abundant cell-free microRNAs (miRNAs) are stably present in numerous human body fluids, including serum, plasma, milk, saliva and urine^7^,^8^,^9^,^10^,^11^. These extracellular miRNAs may reflect molecular changes in the cells from which they are derived and can therefore serve as diagnostic indicators of various diseases such as cancers, infectious diseases and metabolic diseases^12^,^13^,^14^. Interestingly, abundant miRNAs are also detected in human seminal plasma, and these seminal plasma miRNAs may serve as noninvasive biomarkers for diagnosing male infertility^4^,^15^. However, because miRNAs are present in a variety of tissues and organs, the source of seminal plasma miRNAs is elusive; these miRNAs may be secreted from many cell types and tissues. Thus, seminal plasma miRNAs may not provide firsthand information about gene disorders and dysregulation in the male reproductive system. Unlike ubiquitous miRNAs, piwi-interacting RNAs (piRNAs), another type of small RNA, are expressed mainly in pachytene spermatocytes and spermatids in the testes of mammals^16^,^17^,^18^,^19^. piRNAs are longer (26–31 nucleotides) than miRNAs (18–24 nucleotides) and bind to piwi-family proteins. Currently, the major reported function of piRNAs is to provide an elaborate system that protects the germline and gonadal somatic cells against harmful expression of transposable elements and therefore stabilizes male germ cell formation^20^,^21^. piRNAs are produced specifically and abundantly in pachytene spermatocytes and round spermatids and seem to play more important and direct roles in spermatogenesis as well as male infertility^22^. Consequently, piRNAs may be secreted into the seminal plasma in a more straightforward manner than miRNAs and being more tightly correlated with infertility. In this study, we systematically and comprehensively characterized piRNA profiles in the seminal plasma of infertile and fertile males. We also evaluated the diagnostic value of the markedly altered piRNAs in the seminal plasma as an additional tool for male infertility.

## Results

### Profiling seminal plasma piRNAs by high-throughput sequencing

**Figure 1:**
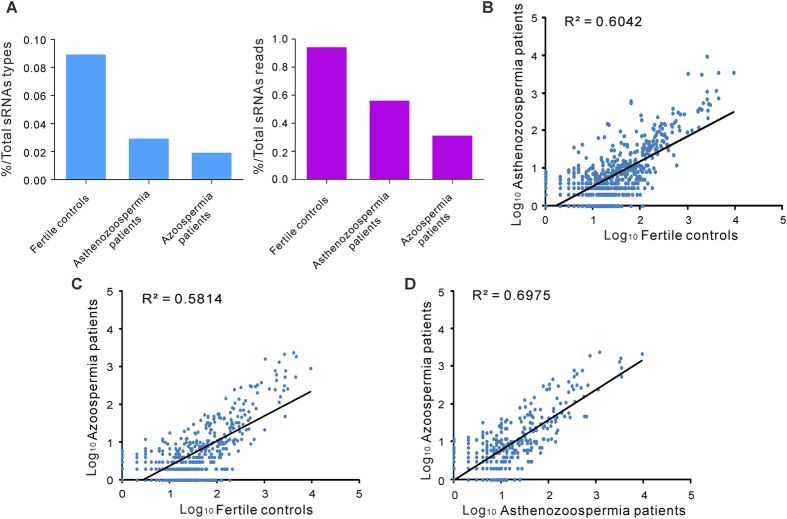
High-throughput sequencing of piRNAs in seminal plasma from infertile patients and healthy controls. (**A**) Total types and sequencing reads of piRNAs were gradually decreased from the seminal plasma of healthy controls to asthenozoospermia patients and then to azoospermia patients. (**B**–**D**) Pearson’s correlation scatter plot of seminal plasma piRNA levels between healthy controls and asthenozoospermia patients (**B**), between healthy controls and azoospermia patients (**C**), and between asthenozoospermia patients and azoospermia patients (**D**).

First, the expression of piRNAs in seminal plasma was screened to identify significantly altered piRNAs between infertile and fertile males. Small RNA was extracted from pooled seminal plasma samples of fertile healthy controls, asthenozoospermia patients and azoospermia patients (each pooled from approximately 20 individuals), and equal concentrations of small RNA were analyzed using Illumina high-throughput sequencing. After masking adaptor sequences and removing short and low-quality reads, a total of 10,641,029; 7,507,768 and 7,287,270 reads of small RNAs within 10 to 45 nucleotides were obtained in the seminal plasma of healthy controls, asthenozoospermia patients and azoospermia patients, respectively. Analysis of the length distribution revealed that all three seminal plasma samples contained a number of small RNAs with sizes that were consistent with the common size of miRNAs (18–24 nucleotides) and piRNAs (26–31 nucleotides) (Supplementary Fig. 1). Next, bioinformatics tools were employed for sorting and evaluating small RNA species and sequencing frequencies. Multiple and heterogeneous small RNA species were identified in seminal plasma, including miRNAs, piRNAs, rRNAs and tRNAs (Supplementary Table 1). Generally, piRNAs occupied approximately 0.05% of total small RNA types and 0.5% of total small RNA amounts (sequencing reads) in seminal plasma. Of the 32,826 piRNAs that were annotated in NCBI database, 3200, 1339 and 743 piRNAs were detected in the seminal plasma of healthy controls, asthenozoospermia patients and azoospermia patients, respectively. Moreover, their levels (types and sequencing reads) gradually decreased from the seminal plasma of healthy controls to asthenozoospermia patients and then to azoospermia patients (Fig. 1A). Consistent with the finding that the piRNAs exhibit a strong bias for uridine at the 5′ end^21^, the bias for a 5′ uridine was also observed in seminal plasma piRNAs. Comparison of the seminal plasma piRNA profiles revealed a global reduction of seminal plasma piRNAs in asthenozoospermia and azoospermia patients relative to healthy controls. These comparisons were analyzed using Pearson’s correlation scatter plots, in which the square of Pearson’s correlation coefficient (R^2^) values was 0.6042 between the asthenozoospermia group and the control group (Fig. 1B) and 0.5814 between the azoospermia group and the control group (Fig. 1C). Asthenozoospermia and azoospermia patients shared a relatively similar seminal plasma piRNA profile, and the R^2^ value for the two groups was 0.6975 (Fig. 1D). We next narrowed down the list of piRNAs to be used as biomarkers of male infertility. The criteria for selecting piRNAs were as follows: 1) if sequencing reads are larger than 1000 in the fertile control group, piRNAs should have at least a 3-fold difference in expression between the infertile patient groups and the fertile control group; and 2) if sequencing reads are between 100 and 1000 in the fertile control group, piRNAs should have at least a 10-fold difference in expression between the infertile patient groups and the fertile control group. Consequently, 61 piRNAs that met the inclusion criteria were chosen for further analysis (Supplementary Table 2).

### Validation of piRNAs in seminal plasma by qRT-PCR and northern blotting analysis

Subsequently, a TaqMan probe-based quantitative RT-PCR (qRT-PCR) assay using individual samples was performed to validate the presence of piRNAs in human seminal plasma. One hundred microliters of seminal plasma was used for RNA extraction, and 8 representative piRNAs (piR-31068, piR-43771, piR-31925, piR-43773, piR-30198, piR-55272, piR-30841 and piR-30495) were assessed by a qRT-PCR assay. To determine the specificity of each primer set, no-template controls for each piRNA were assessed simultaneously. All piRNAs were consistently and efficiently amplified, and the no-template controls amplified at a much higher cycle range than the piRNAs, suggesting that these 8 piRNAs could be readily detected by qRT-PCR in seminal plasma (Supplementary Fig. 2A). The cycle threshold (C_T_) values of some other piRNAs, such as piR-40511, piR-33043 and piR-40978, could not be distinguished from the C_T_ values of the corresponding no-template controls, suggesting that these piRNAs could not be accurately measured by the qRT-PCR assay. Additionally, the levels of piR-31068 and piR-31925 in different volumes of seminal plasma were characterized using the qRT-PCR assay. The qRT-PCR assay showed excellent linearity between the seminal plasma volume and C_T_ value, with the R^2^ value being 0.9903 for piR-31068 and 0.9911 for piR-31925, respectively (Supplementary Fig. 2B). The results suggest that piRNAs in as little as 2 μL of seminal plasma can be efficiently detected and reliably compared across multiple samples. Furthermore, to obtain the absolute concentrations of piRNAs in seminal plasma, each synthetic single-stranded piRNA was serially diluted and assessed using the qRT-PCR assay to generate a standard curve. Decreasing the concentrations of piRNAs led to corresponding increases in the mean C_T_ values, and each piRNA had a R^2^ > 0.99 (Supplementary Fig. 2C). The concentrations of piRNAs in seminal plasma were calculated based on the standard curve.

Next, TA cloning combined with sequencing was performed to confirm the qRT-PCR results. RNA was isolated from seminal plasma samples pooled from healthy controls and infertile patients and subjected to qRT-PCR to amplify piR-31068 and piR-31925, and the amplified products were ligated into a TA vector and sequenced. The sequences of the products were completely identical to the sequences of piR-31068 and piR-31925 (Supplementary Fig. 3A). In addition, northern blotting was used to validate the presence of piRNAs in seminal plasma. In this assay, digoxigenin-labeled oligonucleotide probes containing locked nucleic acids allow for the sensitive and highly specific detection of small RNAs^23^. First, synthetic single-stranded piR-31068 was serially diluted and assessed using northern blotting. The results showed that piR-31068 could be detected at levels greater than 0.01 pmol. Then, RNA isolated from a seminal plasma mixture of healthy controls and infertile patients was assessed. The results of northern blotting showed that piR-31068 was readily detected in the seminal plasma mixture (Supplementary Fig. 3B). For use as a fingerprint in clinical tests, piRNAs should be stable in seminal plasma for reasonable periods of time and preferably resistant to harsh conditions, thereby allowing for routine processing of clinical samples. We therefore investigated the stability of piRNA in seminal plasma stored for extended periods of time. Incubating seminal plasma at room temperature for up to 24 h had minimal effects on the concentrations of piR-31068, piR-31925 and piR-30198 in seminal plasma, demonstrating that these piRNAs are sufficiently stable in seminal plasma (Supplementary Fig. 3C).

### Selection of significantly altered seminal plasma piRNAs as molecular biomarkers for male infertility

**Figure 2:**
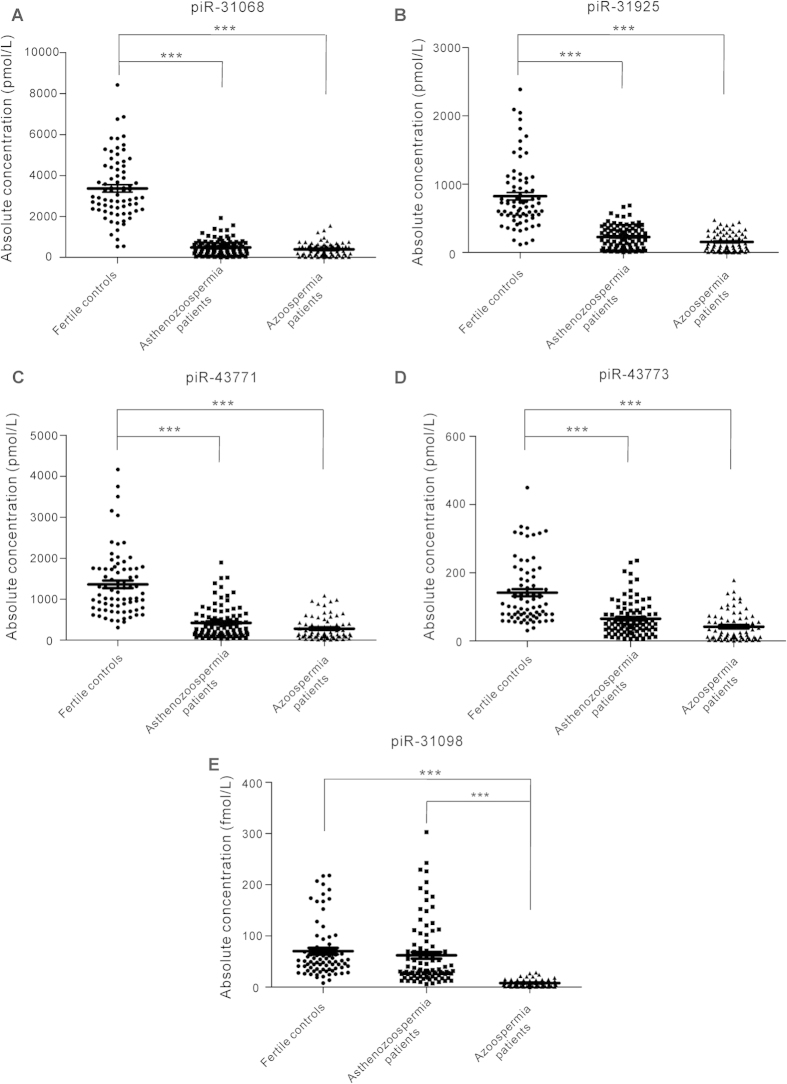
Differential presence of the seminal plasma piRNAs between the infertile patients and healthy controls. (**A**–**E**) The absolute concentrations of piR-31068, piR-31925, piR-43771, piR-43773 and piR-30198 were determined by qRT-PCR in the seminal plasma of asthenozoospermia patients (n = 94) and azoospermia patients (n = 72) compared with those in the seminal plasma of healthy controls (n = 74).

To identify the differentially expressed seminal plasma piRNAs as infertility fingerprints, we performed qRT-PCR analysis of the 61 candidate piRNAs with two sets of individual seminal plasma samples from 74 healthy controls, 94 asthenozoospermia patients and 72 azoospermia patients. Table 1 summarizes the demographic characteristics and semen parameters for all of the participants. There were no significant differences in demographic characteristics among the fertile and infertile males (Table 1). The 61 candidate piRNAs were first measured by qRT-PCR in the training set including 16 healthy controls, 20 asthenozoospermia patients and 20 nonobstructive azoospermia patients. In this phase, only those piRNAs with a mean change >2.0-fold and a *P*-value < 0.01 for comparisons of either of the patient groups and the control group were retained. With these criteria, a panel of 5 piRNAs (piR-31068, piR-31925, piR-43771, piR-43773 and piR-30198) was generated (Table 2). The concentrations of 4 piRNAs (piR-31068, piR-31925, piR-43771 and piR-43773) were significantly decreased in the asthenozoospermia patients and in the azoospermia patients compared with the controls, whereas the concentration of piR-30198 was decreased only in the azoospermia patients compared with the controls. To verify the accuracy and specificity of these 5 piRNAs as an infertility signature, we further assessed their levels in another independent sample set (validation set) consisting of 58 healthy controls, 74 asthenozoospermia patients and 52 azoospermia patients. The trend of alteration of the 5 piRNAs was concordant between the training set and the validation set (Table 2). The differential presence of these 5 piRNAs between the infertile patients and healthy controls is shown in Fig. 2. In summary, as a result of this multiphase test, a profile of 5 seminal plasma piRNAs was selected to be the potential signature for male infertility.

### Diagnostic accuracy of the selected 5 piRNAs as an infertility fingerprint

**Figure 3:**
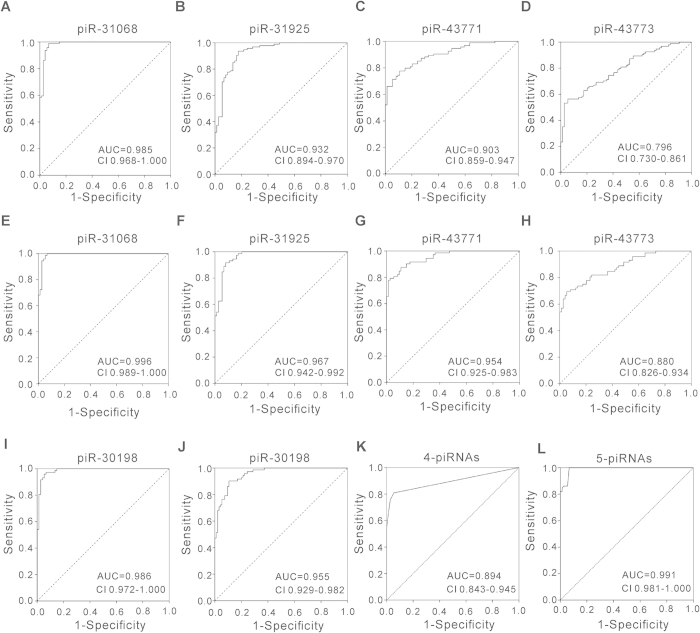
ROC curve analysis. (**A**–**D**) ROC curve for the individual piRNAs piR-31068, piR-31925, piR-43771 and piR-43773 to separate asthenozoospermia patients from healthy controls. (**E**–**I**) ROC curve for the individual piR-31068, piR-31925, piR-43771, piR-43773 and piR-30198 to separate azoospermia patients from healthy controls. (**J**) ROC curve for the individual piR-30198 to separate azoospermia patients from asthenozoospermia patients. (**K**) ROC curve of the 4-piRNA signature (piR-31068, piR-31925, piR-43771 and piR-43773) to separate asthenozoospermia patients from healthy controls. (**L**) ROC curve of the 5-piRNA signature (piR-31068, piR-31925, piR-43771, piR-43773 and piR-30198) to separate azoospermia patients from healthy controls.

To evaluate the usefulness of the selected 5 piRNAs in discriminating infertile patients from healthy controls, we performed receiver-operating characteristic (ROC) curve analysis. ROC curve analysis revealed that 4 piRNAs (piR-31068, piR-31925, piR-43771 and piR-43773) could serve as valuable biomarkers for distinguishing asthenozoospermia patients from healthy controls, with the AUC (the area under the ROC curve) being 0.985, 0.932, 0.903 and 0.796, respectively (Fig.  3A–D). Likewise, the ROC curves also indicated that 5 piRNAs (piR-31068, piR-31925, piR-43771, piR-43773 and piR-30198) could accurately discern azoospermia patients from healthy controls, with the AUC being 0.996, 0.967, 0.954, 0.880 and 0.986, respectively (Fig.  3E–I). Moreover, while piR-31068, piR-31925, piR-43771 and piR-43773 could not distinguish asthenozoospermia patients from azoospermia patients, piR-30198 could serve as an azoospermia-specific biomarker with an AUC of 0.955 (Fig. 3J). The results suggest that the diagnostic potential of these 5 piRNAs to distinguish asthenozoospermic and azoospermic individuals from healthy controls was high.

### Separation of infertile patients from fertile controls by risk score analysis

To further evaluate the diagnostic value of the piRNA signatures in distinguishing infertile patients from fertile controls, we performed a risk score analysis on the data set and employed this risk scoring method to predict infertile patients and fertile controls. First, the risk score equation (1) was used to calculate the risk scores for all samples in the training set. Samples were ranked according to their risk scores and then divided into a high-risk group, representing the predicted infertile patients, or a low-risk group, representing the predicted fertile controls, using an optimal cutoff value (at this cutoff, the value of sensitivity + specificity is maximal). At the cutoff value of 3.57 calculated from the 4-piRNA signature (piR-31068, piR-31925, piR-43771 and piR-43773), only 2 of the fertile controls had a risk score >3.57, whereas 18 of the 20 asthenozoospermia patients exhibited a risk score >3.57 (Table 3). Second, the risk score formula, using the same cutoff value, was used to calculate the risk score for samples from the validation set. Out of 74 asthenozoospermia patients and 58 fertile controls from the validation set, none of controls and 21 patients were incorrectly predicted by this risk scoring method (Table 3). Similarly, when the cutoff value of 5.05 calculated from the 5-piRNA signature (piR-31068, piR-31925, piR-43771, piR-43773 and piR-30198) was used to analyze the sample set of azoospermia patients and fertile controls, only 1 control and 1 patient in the training set and 10 patients in the validation set were incorrectly predicted (Table 3). Furthermore, we integrated the 4-piRNA signature (for asthenozoospermia) and the 5-piRNA signature (for azoospermia) into a single biomarker using the risk score functions and evaluated the diagnostic accuracy of the piRNA signatures as infertility fingerprint. As expected, we obtained an AUC value of 0.894 by combining piR-31068, piR-31925, piR-43771 and piR-43773 for differentiating asthenozoospermia patients from healthy controls (Fig. 3K) and generated a larger AUC value of 0.991 by combining piR-31068, piR-31925, piR-43771, piR-43773 and piR-30198 for differentiating azoospermia patients from healthy controls (Fig. 3L). Finally, we explored the correlation between seminal plasma piRNA levels with clinical features. No obvious correlation was observed when the risk scores of asthenozoospermia patients and healthy controls were stratified by age, sperm density and semen volume, while the risk score value was inversely correlated with the sperm viability (Supplementary Fig. 4). The results suggest a strong relationship between the abnormal piRNA expression and the low sperm viability in infertile patients.

## Discussion

In this study, we found that piRNAs were enriched in seminal plasma, and, for the first time, we demonstrated by sequencing-based global piRNA analysis and by individual qRT-PCR confirmation that the concentrations of seminal plasma piRNAs are altered in infertile patients with azoospermia and asthenozoospermia. Furthermore, ROC curve analysis and risk score analysis revealed a strong relationship between the seminal plasma piRNAs and male infertility, suggesting that the seminal plasma concentrations of piRNAs can accurately distinguish infertile patients from fertile controls.

Currently, the molecular basis of male infertility remains largely unknown, and the role of piRNAs in infertility has not been well documented. Many studies have revealed the critical roles of piRNAs in spermatogenesis, including posttranscriptional regulation of protein-coding genes and the repression of retrotransposons^16^,^18^,^21^. For example, piRNAs are involved in regulating the processes of meiosis and post-meiosis of male germ cell development. A recent study also revealed the siRNA-like function of the piRNA machinery in mouse testes and its central role in male germ cell development and maturation^24^. In addition, piRNAs have an antisense orientation to transposon transcripts and can induce silencing by hybridizing with them. Due to the diverse and pivotal functions of piRNAs in the male reproductive system, dysregulation and dysfunction of piRNAs often lead to male infertility. In this study, we have identified piR-31068, piR-31925, piR-43771, piR-43773 and piR-30198 as molecular biomarkers for male infertility. Further investigations should focus on the biological roles of these piRNAs in spermatogenesis and their association with male infertility, which may provide some pathophysiological clues for the molecular mechanisms underlying this disease.

A crucial requirement for establishing seminal plasma piRNAs as novel infertility indicators is to clarify their source. In this study, high-throughput sequencing technology detected high concentrations of cell-free small RNAs in seminal plasma. One may speculate that these small RNAs are primarily derived from the apoptosis of male germ cells, a key feature of functional spermatogenesis. According to this model, piRNAs leak passively into seminal plasma from apoptotic or broken male germ cells (particularly pachytene spermatocytes and round spermatids). However, a recent study comprehensively characterized the extracellular RNA profile in human semen and found that a substantial amount of small RNAs (including miRNAs, Y RNAs and tRNAs) were contained and protected within seminal exosomes^25^, a type of lipid-bound microparticles originating from multiple cellular sources in the male genital tract. This study even identified a majority of small RNA within the exosomal fraction of seminal plasma rather than the exosome-depleted supernatant^25^. Thus, active secretion from the male germ cells through seminal exosomes likely represents the main source of seminal plasma piRNAs. More importantly, because exosomes can function as the carriers of RNAs from donor cells to recipient cells, piRNAs in seminal exosomes may potentially be delivered to the recipient cells as regulatory signals. In our study, we found that the types and levels of piRNAs were gradually decreased from the seminal plasma of healthy controls to asthenozoospermia patients and then to azoospermia patients. Furthermore, piRNA level in seminal plasma was found to be correlated with sperm viability. The results strongly indicate a tight correlation between the seminal plasma piRNA levels and the function of male germ cells.

In this study, surveying piRNAs in seminal plasma by high-throughput sequencing technology detected little piRNA accumulation (approximately 0.5% of total small RNA sequencing reads); in contrast, miRNAs were approximately 5% of total small RNA sequencing reads in seminal plasma. However, the qRT-PCR results showed that the concentration of piRNAs is much higher in seminal plasma (approximately 1–10000 pmol/L); in contrast, miRNAs is less enriched in seminal plasma (approximately 10–2000 fmol/L), according to our previous study^4^. This inconsistency may be due to the 2′-O-methyl modification in piRNAs. Because the 2′-O-methyl modification of 3′-ends of piRNAs results in decreased efficiency of adaptor ligation, the sequencing procedure is biased against piRNA compared with non-modified small RNAs^26^. That is, if a sample is a mixture of 2′-O-methyl piRNA and 2′-OH small RNA, ligations would favor capture of the 2′-OH small RNA. It is therefore not surprising that the sequencing reads of piRNAs are masked in the large ocean of small RNAs during sequencing of seminal plasma. To efficiently measure the genuine abundance of piRNAs, a strategy of oxidized deep sequencing rather than routine deep sequencing may be required. In this method, piRNAs with 2′-O-methylated 3′-ends are resistant to periodate oxidation and are therefore retained and specifically identified, whereas other small RNAs with unmodified 3′-ends are oxidized and fail to be detected. As a result, in periodate-treated RNAs, an increase in relative abundances of human piRNAs will likely be observed.

In summary, we have defined a distinctive seminal plasma piRNA signature in infertile patients with azoospermia or asthenozoospermia. Moreover, our findings may provide additional information regarding the molecular mechanisms of male infertility.

## Materials and Methods

### Patients and controls

All samples were collected according to protocols approved by the Medical Ethics Committee from Nanjing University, Jinling Hospital and Nanjing Drum Tower Hospital. Fertile male volunteers and all patients signed informed consent for the collection and use of their samples for this study. This study enrolled 211 infertile Chinese men who had failed to achieve conception after a period of >2 years and were referred to the Reproductive Laboratory of Jinling Hospital and Drum Tower Hospital in Nanjing for semen analysis. The fertile control samples were from 91 fertile male volunteers who had fathered a child in a spontaneous conception within the previous 1–2 years, and these men were not receiving any type of medical treatment during the period of the study. The demographic characteristics of infertile patients and healthy controls are listed in Table 1.

### Sample preparation

Semen samples were collected by masturbation into a 15 mL centrifuge tube (Corning) after 3–5 days of sexual abstinence and liquefied for 30 min at 37 °C. After physical examination of the ejaculate, smears of undiluted semen were prepared after liquefaction for assessment of sperm morphology according to the World Health Organization (WHO) criteria^5^. The 211 infertile men samples were subdivided according to their spermiograms: asthenozoospermia (n = 118) or nonobstructive azoospermia (n = 93). Seminal plasma was obtained by centrifuging semen samples at room temperature at 3000 rpm for 5 min, and the supernatant was then collected and stored at −80 °C until analysis.

### Illumina high-throughput sequencing

The sequencing procedure was conducted as previously described^4^,^27^,^28^,^29^,^30^. Briefly, total RNA was extracted from 5 mL of seminal plasma using TRIzol Reagent (Takara, Dalian, China) according to the manufacturer’s instructions. After polyacrylamide gel electrophoresis (PAGE) purification of RNA molecules smaller than 45 nucleotides, the 5′- and 3′-RNA adaptors were ligated to the 5′ and 3′ ends of the RNAs. The small RNA molecules were then transcribed to single-stranded cDNA and amplified using the adaptor primers. The PCR products were supplied for the cluster generation and sequenced using HiSeq™ 2000 System (TruSeq SBS KIT-HS V3, Illumina) at Beijing Genomics Institute (BGI, Shenzhen, China). Image files generated by the sequencer were processed to produce digital-quality data. The subsequent procedures included summarizing data production, evaluating sequence quality and depth, calculating small RNA length distribution and collecting clean reads. The final clean reads were obtained by getting rid of short reads (<10 nucleotides) and contaminant reads (including reads with 5′ primer contaminants or poly A and reads without 3′ primer). After masking the adaptor sequences, the clean reads were aligned to the reference human (*Homo sa*piens) genome. The sequences that correspond to known piRNAs were determined by perfect sequence matching to the NCBI database (http://www.ncbi.nlm.nih.gov). miRNA sequences were determined by matching to the miRNA database (miRBase 19.0), and other small RNAs (rRNA, tRNA and snoRNA) were annotated by blasting against the Rfram database (http://sanger.ac.uk/software/Rfam). Finally, the total sequencing frequency of each sample was adjusted to an equal scale of 10,641,029 (same as the total reads of healthy controls). All data have been uploaded to the GEO database (Accession number: GSE78131).

### RNA isolation and qRT-PCR assays

Total RNA was extracted from 100 μL of seminal plasma using TRIzol Reagent (TaKaRa, Dalian). Briefly, 100 μL of seminal plasma was mixed with 1 mL of TRIzol Reagent (TaKaRa) in a 1.5 mL microcentrifuge tube (RNase-free, Axygen). The mixture was vortex-mixed vigorously for 10 s and then added to 20 μL of synthetic MIR2911. The mixture was further vortex-mixed vigorously for 20 s, and 200 μL of chloroform was then added. The mixture was vortex-mixed vigorously for 30 s, incubated on ice for 10 min, and then centrifuged at 16,000 g for 20 min at 4 °C. The supernatant was transferred to a new 1.5 mL microcentrifuge tube. Then, the same volume of isopropanol was added to the supernatant, and the tube was inverted 10 times to mix thoroughly. Next, the mixture was stored at −20 °C for 1.5 h and then centrifuged at 16,000 g for 20 min at 4 °C. Subsequently, the RNA pellet was collected, washed once with 75% ethanol and dried for 20 min at room temperature. Finally, the pellet was dissolved in 40 μL of RNase-free water and stored at −80 °C until further analysis.

The qRT-PCR assays were conducted on the LightCycler 480 Real-Time PCR system (Roche) according to the manufacturer’s instructions. Reverse transcriptions were performed as previously described with minor modification. In brief, 2 μL of total RNA was reverse-transcribed to cDNA using AMV reverse transcriptase (TaKaRa, Dalian, China) and a stem-loop RT primer (GenePharma, Shanghai, China). The reaction conditions were as follows: 16 °C for 30 min, 42 °C for 30 min and 85 °C for 5 min. Then, 2 μL of cDNA generated from reverse transcription was used for PCR analysis, and real-time PCR was performed using hydrolysis probes of piRNAs (custom-made probes from GenePharma) on a Roche LightCycler 480 PCR system. The reactions were incubated in a 96-well optical plate at 95 °C for 5 min, followed by 40 cycles of 95 °C for 15 s and 62 °C for 1 min. All reactions were run in triplicate. After the reaction, the C_T_ values were determined using the fixed threshold settings, and the mean C_T_ values were determined from triplicate PCRs.

### Northern blotting analysis

A sensitive non-radioactive northern blot method was performed to detect seminal plasma piRNAs as previously described^31^. Total RNA was extracted from 50 μL and 625 μL of seminal plasma mixture using the TRIzol Reagent (TaKaRa). Northern blot analysis was conducted using piRNA Detection Probes with DIG labeling (GenScript Corporation) and a DIG luminescent detection kit (Roche, Indianapolis, IN, USA) according to the manufacturer’s instructions.

### Risk score analysis

Risk score analysis was performed to evaluate the association between male infertility and the piRNA expression levels. The risk score of each piRNA in the training set, denoted as “*s*”, was set as 1 if the expression level was lower than the lower 5% reference interval limit for the corresponding piRNA level in controls and as 0 if otherwise. Taking into account the association of each piRNA with infertility risk, each patient was assigned a risk score function (RSF) according to a linear combination of the piRNA expression levels. The RSF for sample *i* using the information from the piRNA level was:


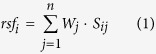


In the equation (1), *s*_*ij*_ is the risk score for piRNA *j* on sample *i*, and W*j* is the weighting given to the risk score of piRNA *j*. To determine the *W·s*, *n* univariate logistic regression models were fitted using the disease status with each of the risk scores. The regression coefficient of each risk score was used as the weight to indicate the contribution of each piRNA to the RSF. Frequency tables and ROC curves were then used to evaluate the diagnostic effectiveness of the piRNA profile as infertility biomarkers and to find an appropriate cutoff point. Then the cutoff values determined in the training sample set were applied in the validation sample set.

### Statistical analysis

Statistical analysis was performed with SPSS 22.0 software. piRNA concentration data were represented as the means ± SEs. The t-test was used to compare differences of seminal plasma piRNA between infertile groups and the control group. A *P*-value < 0.01 was considered statistically significant. The ROC curve was constructed to evaluate the diagnostic effectiveness of the selected piRNAs for male infertility.

## Additional Information

**How to cite this article**: Hong, Y. *et al*. Systematic characterization of seminal plasma piRNAs as molecular biomarkers for male infertility. *Sci. Rep*. **6**, 24229; doi: 10.1038/srep24229 (2016).

## Supplementary Material

Supplementary Information

## Figures and Tables

**Table 1:** Demographic and clinical features of the infertile patients and fertile controls^a^.

Variables (screening set)	Fertile controls (n = 17)	Asthenospermia patients (n = 24)	Azoospermia patients (n = 21)	*P*-Value^b^	*P*-Value^c^	*P*-Value^d^
Average age, years	27.43 (4.34)	30.21 (7.02)	31 (7.51)	0.7313	0.0746	0.1237
Sexual abstinence time, days	4 (0.77)	4.04 (0.81)	3.94 (0.84)	0.6994	0.8226	0.861
Semen parameters						
pH	7.34 (0.19)	7.33 (0.16)	7.31 (0.17)	0.7208	0.5918	0.8061
Total volume, mL	3.48 (1.74)	3.75 (2.06)	3.24 (1.12)	0.3558	0.6252	0.6353
Sperm parameters						
Sperm density, × 10^6^/mL	78.07 (54.19)	57.82 (38.16)	0	0.2104	1.28 × 10^−7^	2.86 × 10^−8^
Sperm viability, %	69.73 (8.58)	12.21 (6.58)	0	5.69 × 10^−28^	3.04 × 10^−41^	1.09 × 10^−10^
a + b^e^	53.43 (2.65)	8.67 (4.26)	0	7.35 × 10^−22^	7.72 × 10^−27^	9.78 × 10^−13^
**Variables (training and validation sets)**	**(n = 74)**	**(n = 94)**	**(n = 72)**			
Average age, years	30.17 (4.6)	29.77 (5.74)	29.27 (4.18)	0.6644	0.2857	0.5925
Sexual abstinence time, days	4.02 (0.75)	4.03 (0.72)	3.90 (0.66)	0.9538	0.2774	0.2678
Semen parameters						
pH	7.29 (0.17)	7.32 (0.17)	7.28 (0.12)	0.2195	0.6945	0.0831
Total volume, mL	3.92 (1.48)	4.16 (1.70)	3.78 (1.02)	0.392	0.5849	0.1563
Sperm parameters						
Sperm density, × 10^6^/mL	61.04 (25.26)	57.31 (26.01)	0	0.5342	7.62 × 10^−33^	2.88 × 10^−27^
Normal sperm morphology, %	15.18 (1.99)	3.03 (3.0)	0	1.32 × 10^−19^	1.03 × 10^−31^	4.98 × 10^−31^
Sperm viability, %	75.01 (8.68)	16.38 (7.9)	0	1.28 × 10^−48^	1.68 × 10^−79^	1.41 × 10^−26^
a^e^	35.39 (12.83)	4.22 (3.86)	0	2.66 × 10^−25^	6.90 × 10^−38^	7.93 × 10^−12^
a + b^e^	61.80 (21.49)	11.99 (6.75)	0	2.96 × 10^−39^	6.03 × 10^−62^	8.95 × 10^−22^

^a^Data are presented as mean (SD).

^b^Fertile controls vs. Asthenozoospermia patients.

^c^Fertile controls vs. Azoospermia patients.

^d^Asthenozoospermia patients vs. Azoospermia patients.

^e^a, rapid progressive motility; ^e^a + b, progressive motility.

**Table 2:** piRNA concentrations in the seminal plasma samples from infertile and fertile males^a^.

piRNA	Fertile controls (pmol/L)	Asthenozoospermia patients (pmol/L)	Azoospermia patients (pmol/L)	*P*-Value^b^	*P*-Value^c^	*P*-Value^d^
Training set						
samples, n	16	20	20			
piR-31068	2153.12 (309.36)	330.24 (115.69)	305.24 (107.49)	9.06 × 10^−7^	5.64 × 10^−7^	0.88
piR-31925	628.2 (106.76)	169.34 (47.25)	124.73 (31.45)	1.75 × 10^−4^	1.92 × 10^−5^	0.44
piR-43771	944.86 (121.26)	327.95 (77.8)	201.27 (48.25)	8.97 × 10^−5^	5.45 × 10^−7^	0.17
piR-43773	91.13 (11.19)	38.31 (7.35)	33.24 (5.91)	2.54 × 10^−4^	2.81 × 10^−5^	0.59
piR-30198	66.52 (15.3) × 10^−3^	56.15 (14.4) × 10^−3^	14.13 (1.7) × 10^−3^	0.63	5.63 × 10^−4^	6.23 × 10^−3^
Validation set						
samples, n	58	74	52			
piR-31068	3700.66 (187.92)	532.79 (39.16)	435.24 (33.13)	4.76 × 10^−38^	9.15 × 10^−31^	0.08
piR-31925	877.99 (63.42)	242.58 (17.52)	164.99 (17.58)	1.65 × 10^−19^	8.79 × 10^−18^	0.003
piR-43771	1475.51 (107.67)	445.21 (46.19)	308.69 (40.4)	1.52 × 10^−16^	2.0 × 10^−16^	0.04
piR-43773	155.54 (12.6)	72.49 (6.06)	45.25 (6.43)	3.32 × 10^−9^	1.59 × 10^−11^	0.003
piR-30198	71.05 (6.97) × 10^−3^	63.66 (7.17) × 10^−3^	5.59 (0.62) × 10^−3^	0.47	1.77 × 10^−14^	4.72 × 10^−10^

^a^piRNA data are presented as the mean (SE).

^b^Fertile controls vs. Asthenozoospermia patients.

^c^Fertile controls vs. Azoospermia patients.

^d^Asthenozoospermia patients vs. Azoospermia patients.

**Table 3:** Risk score analysis of infertile patients and fertile controls.

Risk score analysis of asthenozoospermia patients and fertile controls				
Score	0–3.75	3.75–14.56	PPV^a^	NPV^b^
Training set			0.900	0.875
Fertile controls	14	2		
Asthenozoospermia	2	18		
Validation set			1.000	0.734
Fertile controls	58	0		
Asthenozoospermia	21	53		
**Risk score analysis of azoospermia patients and fertile controls**				
**Score**	**0–5.05**	**5.05–19.04**	**PPV**^a^	**NPV**^b^
Training set			0.950	0.938
Fertile controls	15	1		
Azoospermia	1	19		
Validation set			1.000	0.853
Fertile controls	58	0		
Azoospermia	10	42		

^a^PPV, positive predictive value.

^b^NPV, negative predictive value.
