# Supplementary material for: Systematic characterization of seminal plasma piRNAs as molecular biomarkers for male infertility
Source: Sci Rep. 2016 Apr 12;6:24229. doi: 10.1038/srep24229 (PMC4828650; doi:10.1038/srep24229)
Supplement: Supplementary Information [file srep24229-s1.doc]

**Systematic characterization of seminal plasma piRNAs as molecular biomarkers for male infertility**

Yeting Hong, Cheng Wang, Zheng Fu, Hongwei Liang, Suyang Zhang, Meiling Lu, Wu Sun, Chao Ye, Chen-Yu Zhang, Ke Zen, Liang Shi, Chunni Zhang, Xi Chen

**Supplementary Figures**

**
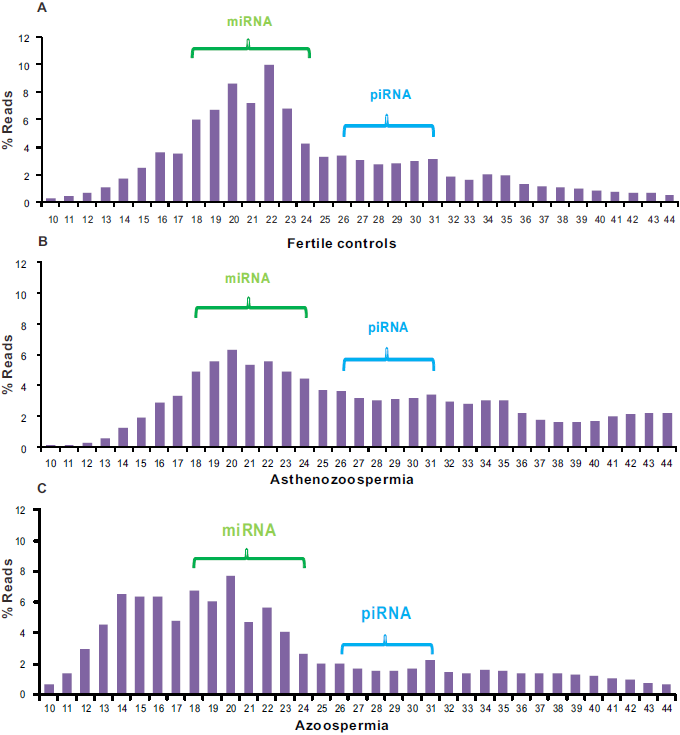
**

**Supplementary Figure 1.** The distribution of small RNAs of various lengths (10-44 bp) measured by high-throughput sequencing in seminal plasma from healthy controls, asthenozoospermia cases and azoospermia cases.


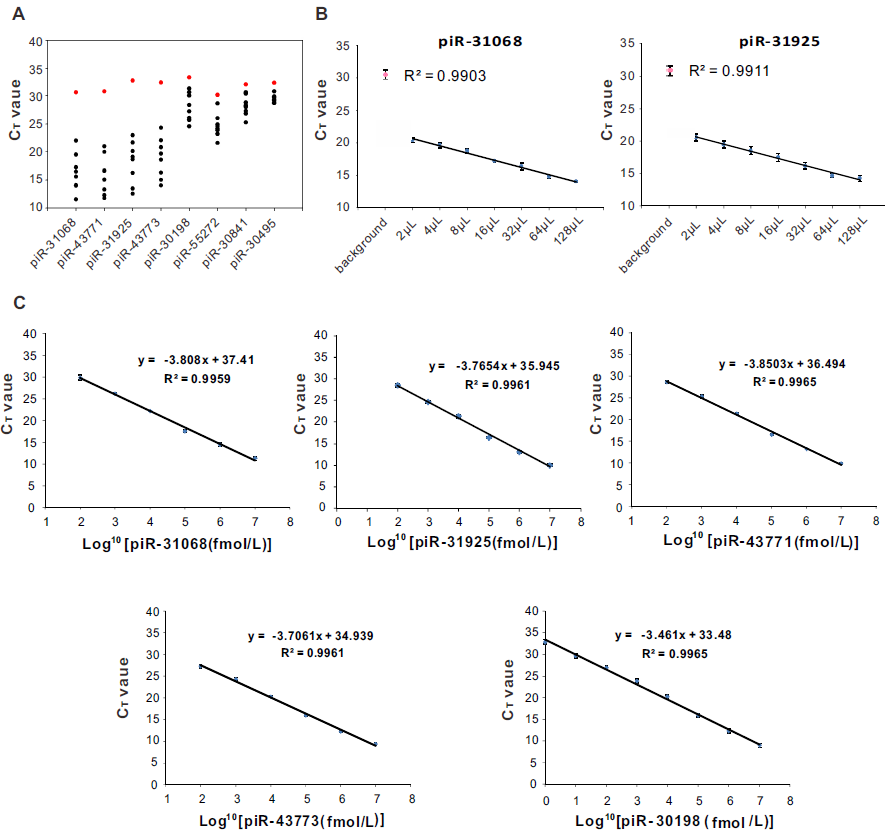


**Supplementary Figure 2. Dynamic range and sensitivity of the qRT-PCR assay for measuring piRNAs. (A)** The raw C_T_ values of piRNAs determined by qRT-PCR assay. One hundred microliters of seminal plasma was used for RNA extraction, and several piRNAs were assessed by a qRT-PCR assay (black dots). An assay using water instead of RNA for the qRT-PCR assay was included as a no-template control (red dots). **(B)** Correlation of the seminal plasma volume to the C_T_ values. Total RNA was extracted from different volumes of seminal plasma mixture ranging from 2 to 128 μL. The levels of piR-31068 and piR-31925 were assessed by qRT-PCR. The resulting C_T_ values were plotted versus the seminal plasma volume used for RNA extraction. An assay using water instead of RNA for qRT-PCR assay was included as a negative control. **(C)** Standard curves of piRNAs. Synthetic single-stranded piRNAs were serially diluted over several orders of magnitude and were assessed by the qRT-PCR assay. The resulting C_T_ values were plotted versus the concentration of input piRNA to generate a standard curve.


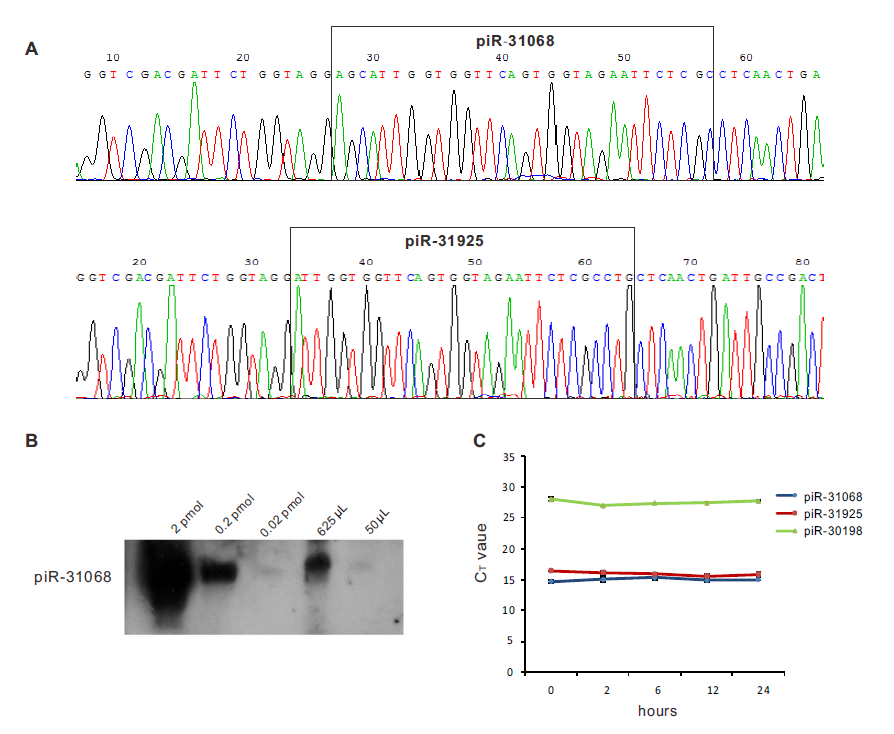


**Supplementary Figure 3. Validation of piRNAs in seminal plasma by TA-cloning/sequencing and northern blotting. (A)** TA cloning and sequencing of amplified PCR products. Small RNA isolated from the seminal plasma mixture of infertile patients and healthy controls was subjected to qRT-PCR to amplify piR-31068 and piR-31925, and then the amplified products were ligated into a TA-vector and sequenced. **(B)** Northern blot analysis of piR-31068 in different volumes (625 vs. 50 μL) of the seminal plasma mixture of infertile patients and healthy controls. Synthetic single-stranded piR-31068 was serially diluted (from 2 to 0.02 pmol) and assessed via northern blotting simultaneously as a positive control. **(C)** The stability of piRNAs in seminal plasma after extended storage. Seminal plasma samples were equally divided and stored at room temperature for 0, 2, 6, 12 or 24 h. For each time point, total RNA was isolated, and piR-31068, piR-31925 and piR-30198 were measured by qRT-PCR assay. Storage at room temperature for 24 h yielded no apparent increases in C_T_ values.


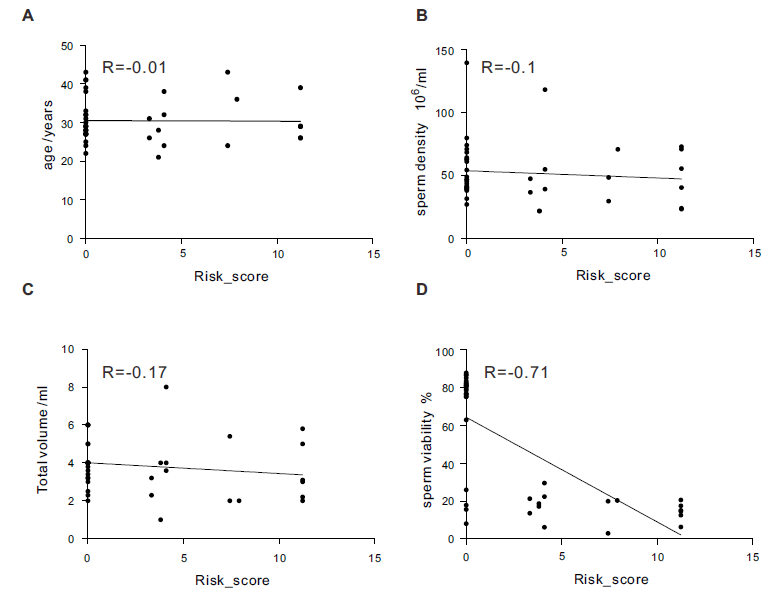


**Supplementary Figure 4. Correlation between seminal plasma piRNA levels with clinical features.** Risk score values of asthenozoospermia patients (n=20) and healthy controls (n=20) were correlated with the clinical features of age (A), sperm density (B), semen volume (C) and sperm viability (D). R value represents the Pearson’s correlation coefficient.

**Supplementary Tables**

**Supplementary Table 1. Distribution of small RNAs among different categories.**

**Fertile healthy controls**

| **Category** | **Unique small RNA types** | **Percent (%)** | **Total small RNA reads** | **Percent (%)** |
| --- | --- | --- | --- | --- |
| **Total** | 3613088 | 100% | 10641029 | 100% |
| **exon_antisense** | 55707 | 1.54% | 96984 | 0.91% |
| **exon_sense** | 100862 | 2.79% | 162827 | 1.53% |
| **intron_antisense** | 201579 | 5.58% | 281145 | 2.64% |
| **intron_sense** | 289168 | 8% | 416173 | 3.91% |
| **miRNA** | 4123 | 0.11% | 1013162 | 9.52% |
| **piRNA** | 3600 | 0.09% | 101340 | 0.95% |
| **rRNA** | 92887 | 2.57% | 1786029 | 16.78% |
| **repeat** | 902406 | 24.98% | 1482217 | 13.93% |
| **scRNA** | 15463 | 0.43% | 296941 | 2.79% |
| **snRNA** | 10602 | 0.29% | 42662 | 0.40% |
| **snoRNA** | 2789 | 0.08% | 7014 | 0.07% |
| **srpRNA** | 543 | 0.02% | 1603 | 0.02% |
| **tRNA** | 65054 | 1.80% | 1651475 | 15.52% |
| **unann** | 1871905 | 51.92% | 3301457 | 31.03% |

**Asthenozoospermia patients**

| **Category** | **Unique small RNA types** | **Percent (%)** | **Total small RNA reads** | **Percent (%)** |
| --- | --- | --- | --- | --- |
| **Total** | 4630012 | 100% | 7507768 | 100% |
| **exon_antisense** | 74911 | 1.62% | 106813 | 1.42% |
| **exon_sense** | 82330 | 1.78% | 106385 | 1.42% |
| **intron_antisense** | 271593 | 5.87% | 329795 | 4.39% |
| **intron_sense** | 394572 | 8.52% | 494144 | 6.58% |
| **miRNA** | 1950 | 0.04% | 114288 | 1.52% |
| **piRNA** | 1339 | 0.03% | 42901 | 0.57% |
| **rRNA** | 105444 | 2.28% | 575371 | 7.66% |
| **repeat** | 1237566 | 26.73% | 1756611 | 23.40% |
| **scRNA** | 14992 | 0.32% | 81261 | 1.08% |
| **snRNA** | 11047 | 0.24% | 21758 | 0.29% |
| **snoRNA** | 3022 | 0.07% | 6649 | 0.09% |
| **srpRNA** | 509 | 0.01% | 863 | 0.01% |
| **tRNA** | 53324 | 1.15% | 771888 | 10.28% |
| **unann** | 2377413 | 51.35% | 3099041 | 41.28% |

**Azoospermia patients**

| **Category** | **Unique small RNA types** | **Percent (%)** | **Total small RNA reads** | **Percent (%)** |
| --- | --- | --- | --- | --- |
| **Total** | 3190082 | 100% | 7287270 | 100% |
| **exon_antisense** | 39101 | 1.23% | 54419 | 0.75% |
| **exon_sense** | 63003 | 1.97% | 85478 | 1.17% |
| **intron_antisense** | 155197 | 4.86% | 199445 | 2.74% |
| **intron_sense** | 216898 | 6.80% | 284020 | 3.90% |
| **miRNA** | 2847 | 0.09% | 465498 | 6.39% |
| **piRNA** | 743 | 0.02% | 23054 | 0.32% |
| **rRNA** | 61500 | 1.93% | 1225189 | 16.81% |
| **repeat** | 703601 | 22.06% | 1078762 | 14.80% |
| **scRNA** | 11675 | 0.37% | 141546 | 1.94% |
| **snRNA** | 8072 | 0.25% | 22800 | 0.31% |
| **snoRNA** | 2211 | 0.07% | 5162 | 0.07% |
| **srpRNA** | 336 | 0.01% | 735 | 0.01% |
| **tRNA** | 43659 | 1.37% | 916021 | 12.57% |
| **unann** | 1881239 | 58.79% | 2785141 | 38.22% |

**Supplementary Table 2. Selected piRNAs that were significantly downregulated in seminal plasma of infertile patients compared with fertile controls. The values showing in the columns referred to sequencing read counts after normalization.**

| **ID** | **piRNA** | **Fertile controls** | **Asthenospermia** | **Azoospermia** |
| --- | --- | --- | --- | --- |
| DQ588409.1 | piR-55521 | 9278 | 4850 | 1323 |
| DQ575659.1 | piR-43771 | 4521 | 1059 | 2796 |
| DQ588410.1 | piR-55522 | 4494 | 4921 | 809 |
| DQ571592.1 | piR-31704 | 2982 | 687 | 390 |
| DQ575661.1 | piR-43773 | 2782 | 734 | 1145 |
| DQ597972.1 | piR-36038 | 2671 | 488 | 799 |
| DQ570729.1 | piR-30841 | 2663 | 302 | 70 |
| DQ576942.1 | piR-45054 | 2595 | 961 | 460 |
| DQ588582.1 | piR-55694 | 2521 | 1586 | 358 |
| DQ588443.1 | piR-55555 | 2112 | 784 | 239 |
| DQ588359.1 | piR-55471 | 1992 | 510 | 172 |
| DQ598176.1 | piR-36242 | 1748 | 561 | 930 |
| DQ570956.1 | piR-31068 | 1730 | 174 | 663 |
| DQ592931.1 | piR-33043 | 1649 | 495 | 1154 |
| DQ576941.1 | piR-45053 | 1635 | 475 | 361 |
| DQ579193.1 | piR-47305 | 1233 | 149 | 378 |
| DQ588328.1 | piR-55440 | 977 | 132 | 32 |
| DQ598641.1 | piR-36707 | 583 | 17 | 18 |
| DQ572866.1 | piR-40978 | 502 | 31 | 13 |
| DQ588393.1 | piR-55505 | 465 | 132 | 34 |
| DQ588372.1 | piR-55484 | 407 | 58 | 32 |
| DQ585858.1 | piR-52970 | 329 | 207 | 25 |
| DQ588447.1 | piR-55559 | 318 | 113 | 18 |
| DQ595702.1 | piR-61814 | 273 | 52 | 9 |
| DQ576809.1 | piR-44921 | 266 | 58 | 16 |
| DQ578919.1 | piR-47031 | 235 | 34 | 18 |
| DQ592278.1 | piR-59390 | 225 | 51 | 13 |
| DQ576808.1 | piR-44920 | 221 | 78 | 15 |
| DQ595288.1 | piR-61400 | 216 | 38 | 16 |
| DQ586816.1 | piR-53928 | 206 | 11 | 3 |
| DQ571813.1 | piR-31925 | 195 | 14 | 58 |
| DQ588413.1 | piR-55525 | 183 | 164 | 18 |
| DQ572237.1 | piR-40349 | 181 | 4 | 1 |
| DQ578464.1 | piR-46576 | 176 | 27 | 7 |
| DQ588373.1 | piR-55485 | 163 | 47 | 16 |
| DQ570383.1 | piR-30495 | 160 | 11 | 3 |
| DQ575741.1 | piR-43853 | 155 | 61 | 10 |
| DQ588329.1 | piR-55441 | 153 | 31 | 3 |
| DQ601822.1 | piR-39888 | 151 | 13 | 10 |
| DQ595661.1 | piR-61773 | 149 | 69 | 15 |
| DQ588346.1 | piR-55458 | 148 | 51 | 15 |
| DQ588584.1 | piR-55696 | 143 | 6 | 22 |
| DQ596483.1 | piR-34549 | 142 | 17 | 3 |
| DQ593628.1 | piR-33740 | 135 | 78 | 13 |
| DQ595210.1 | piR-61322 | 135 | 11 | 13 |
| DQ571243.1 | piR-31355 | 132 | 17 | 10 |
| DQ578908.1 | piR-47020 | 125 | 51 | 7 |
| DQ571634.1 | piR-31746 | 124 | 7 | 3 |
| DQ573371.1 | piR-41483 | 124 | 4 | 0 |
| DQ588160.1 | piR-55272 | 122 | 4 | 1 |
| DQ589179.1 | piR-56291 | 122 | 31 | 7 |
| DQ598828.1 | piR-36894 | 117 | 14 | 1 |
| DQ588344.1 | piR-55456 | 114 | 7 | 6 |
| DQ578005.1 | piR-46117 | 112 | 7 | 7 |
| DQ570086.1 | piR-30198 | 112 | 1 | 0 |
| DQ588364.1 | piR-55476 | 111 | 48 | 9 |
| DQ576945.1 | piR-45057 | 108 | 16 | 6 |
| DQ586492.1 | piR-53604 | 104 | 13 | 0 |
| DQ577685.1 | piR-45797 | 103 | 7 | 12 |
| DQ588326.1 | piR-55438 | 101 | 14 | 0 |
| DQ593768.1 | piR-33880 | 100 | 3 | 16 |
